# Evaluating potential program cost savings with a single-dose HPV vaccination schedule: a modeling study

**DOI:** 10.1093/jncimonographs/lgae037

**Published:** 2024-11-12

**Authors:** Rose Slavkovsky, Mercy Mvundura, Frédéric Debellut, Teddy Naddumba

**Affiliations:** Center for Vaccine Innovation and Access, PATH, Seattle, WA, USA; Medical Devices and Health Technologies, PATH, Seattle, WA, USA; Center for Vaccine Innovation and Access, PATH, Geneva, Switzerland; Medical Devices and Health Technologies, PATH, Kampala, Uganda

## Abstract

**Background:**

There is limited evidence on the magnitude of the potential program cost savings associated with the World Health Organization–endorsed single-dose schedule for the human papillomavirus (HPV) vaccine. The objective of this analysis was to model the delivery and vaccine procurement cost implications of the new schedule.

**Methods:**

The analysis leveraged primary data during a study evaluating the HPV vaccine delivery costs and operational context in 5 countries (Ethiopia, Guyana, Rwanda, Sri Lanka, and Uganda) implementing a two-dose schedule. To estimate the cost for the single-dose schedule, we adjusted the two-dose schedule cost estimates to account for differences in the frequency of activities, whether activities differed by HPV vaccine dose or session, and differences in relative quantity or storage volume of HPV vaccines delivered. We estimated the cost per dose and cost per adolescent receiving the full (single-dose or two-dose) vaccination schedule in 2019 US dollars from a health system perspective.

**Results:**

Modeled results found that cost per dose would increase under a single-dose schedule, whereas cost per adolescent receiving the full schedule would decrease. The financial cost for vaccine procurement and delivery per adolescent receiving the full schedule ranged from $9.64 (Sri Lanka) to $23.43 (Guyana) under a two-dose schedule and decreased to $4.84 and $12.34, respectively, under a single-dose schedule, reflecting savings up to 50%. For economic costs, the range for a single-dose schedule was $7.86 (Rwanda) to $28.53 (Guyana).

**Conclusion:**

A single-dose HPV vaccination schedule could provide cost savings to immunization programs and enhance program affordability and sustainability.

Human papillomavirus (HPV) vaccination is one of three pillars set out by the World Health Organization (WHO) to accelerate cervical cancer elimination (1). Cervical cancer ranks as the fourth leading cause of cancer mortality in women globally (2), with low- and middle-income countries (LMICs) bearing the greatest share of this burden (3).

The first vaccines to protect against oncogenic HPV types became available in 2006 and were originally licensed for a three-dose schedule (4). In 2014, WHO recommended a two-dose schedule for the primary target population of 9- to 13-year-old girls based on new efficacy data (5). WHO endorsed a single-dose or two-dose HPV vaccination schedule in 2022 (6), as a single dose of HPV vaccine was shown to have comparable efficacy and duration of protection as multiple doses, and a single-dose schedule may also offer program advantages (7). Countries began adopting a single-dose schedule as early as 2022 (8,9) given this evidence on the population health impact and the associated additional benefits such as simplified logistics of delivering fewer doses, the possibility of improved vaccine coverage, and the anticipated cost savings for vaccine procurement (6,7), which comprises a substantial part of HPV vaccination program costs (10). However, there is limited evidence on the ongoing delivery costs to programs implementing a single-dose HPV vaccination schedule.

HPV vaccine delivery cost estimates in the literature are from three- and two-dose schedules, with data collected primarily during pilots, demonstration projects, or the introduction period (10). Only two prior costing studies provided evidence on the changes to delivery costs under a single-dose schedule through modeling a hypothetical single-dose schedule in Tanzania (11) and cost projections for a hypothetical two-dose and one-dose national program in Mozambique (12). These studies indicate that overall program costs are likely to decrease, cost per dose delivered to increase, and cost per fully immunized girl to decrease. We sought to expand this evidence base and provide additional modeled estimates from 5 countries (Ethiopia, Guyana, Rwanda, Sri Lanka, and Uganda) representing three WHO regions until empiric costing data from a single-dose program becomes available.

Building on a recent study that evaluated the operational context and costs of HPV vaccine delivery in 5 countries implementing a two-dose schedule (13), we aimed to estimate the potential cost savings for delivery and procurement costs under a single-dose schedule.

## Methods

This modeling analysis used data from a retrospective HPV vaccine cost of delivery and operational context study conducted in countries implementing a two-dose schedule, which aimed to understand the frequency and intensity of activities and resources used in 2019 for HPV vaccination (13). Our analysis used data from Ethiopia, Guyana, Rwanda, Sri Lanka, and Uganda with sample sizes in each country ranging from 30 to 66 health facilities, 5 to 29 subnational administrative offices, and 1 national office (Supplementary Table 1, available online) (13). Eleven program activities were evaluated, including program planning, social mobilization, training, vaccine collection or distribution and storage, and service delivery (Table 1). Microcosting methods were used to identify, quantify, and value financial and economic resources for each program activity evaluated. The financial and economic costs evaluated to estimate delivery costs are shown in Table 1. Economic costs included financial costs and the opportunity costs of use of existing resources by the HPV vaccination program, which do not incur a financial outlay (14). Opportunity costs were time costs for human resources and the annualized costs of vehicles, cold chain equipment, and incinerators owned by the Ministry of Health. Costs were considered from a health system perspective and did not differentiate between payers.

**Table 1.** Program activities and cost categories evaluated for HPV vaccine delivery and adjustment factors used to estimate vaccine delivery costs under a hypothetical single-dose schedule

Program activity	Cost categories	Adjustments for health facility level calculations	Adjustments for administrative level calculations
Service delivery	Per diems; vehicle rental; fuel, maintenance, and capital cost of vehicles; health worker and non-health worker time	Session-based	n/a
Vaccine collection and storage	Per diems; vehicle rental	Quantity- and dose-based	Quantity- and dose-based
	Fuel, maintenance, and capital cost of vehicles	Session-based	Dose-based
	Capital cost of cold chain equipment; energy costs for cold chain equipment	Volume-based	Volume-based
	Health worker time	Dose-based	n/a
Social mobilization and information, education, and communication (IEC)	Per diems; meeting costs; other costs; non-health worker time	Frequency-based	Frequency-based
	Health worker time	Dose-based	n/a
Program planning and management	Per diems; other costs; non-health worker time	No adjustment	
	Health worker time	Dose-based	n/a
Training	Per diems; venue costs; non-health worker time	Frequency-based	Frequency-based
	Health worker time	Dose-based	n/a
Crisis management	Per diems; other costs; health worker and non-health worker time	Dose-based	Dose-based
Supervision	Health worker time	n/a	Dose-based
Vaccine procurement	Extra cost; health worker time	Dose-based	Dose-based
Estimating demand	Health worker time	Dose-based	n/a
Record keeping	Copies	Session-based	Dose-based
	Health worker time	Dose-based	n/a
Waste management	Fuel costs; other costs	Dose-based	Dose-based
	Incinerator capital costs	Volume-based	Volume-based

n/a = not applicable.

Health facility and program office staff responsible for implementing the HPV vaccination program were interviewed using structured questionnaires. For training and social mobilization activities, respondents were asked whether activities were done for the first (HPV1), second (HPV2), or both HPV vaccine doses. In addition, secondary data were obtained from each country’s immunization program on the number of routine infant vaccine doses delivered at each level of the health system and the number of HPV vaccine doses delivered, disaggregated by HPV1 and HPV2 when available, during the reference year. These data were used to compute quantity-based and volume-based allocation factors for resources shared across the immunization program. Quantity-based allocation considered the total number of HPV vaccine doses as a proportion of all vaccine doses delivered at each level of the health system during the reference period, whereas volume-based allocations considered the cold chain volume (in cm^3^) of HPV vaccines as a proportion of the volume of all vaccine doses delivered during the same period (13). We calculated the mean cost per facility (health facility or program office) for HPV vaccination activities, the cost per dose delivered at each level of the health system, and the cost per dose delivered aggregated across all levels of the health system.

For the modeling analysis, we first calculated 5 adjustment factors using primary and secondary data collected during the completed costing and operational research study (Table 2). The country-specific values for each adjustment factor are listed in Supplementary Table 2 (available online). Using the adjustment factors, we recalculated the financial and economic costs for each of the program activities evaluated at each level of the health system, as shown in Table 1. We then re-estimated the financial and economic cost per dose aggregated across all levels of the health system, using the estimated number of HPV1 doses delivered in our sample as the denominator and excluding procurement costs of vaccines and syringes. We also calculated the cost per adolescent receiving the full schedule under a two-dose and single-dose schedule. Lastly, we estimated the impact of implementing a single-dose schedule on vaccine and syringe procurement costs using the dose-based adjustment factor. The procurement price of HPV vaccine per dose was $4.50 for all countries except for Guyana ($9.58), and syringe costs ranged from $0.03 to $0.06 (Supplementary Table 2, available online). We included the full vaccine price in our calculations, as our health system perspective did not differentiate by payer.

**Table 2.** Adjustment factors to recalculate cost estimates for HPV vaccine delivery costs under a hypothetical single-dose schedule

Adjustment factor	Description	Data source
Dose-based adjustment	Proportion of HPV1 doses delivered among all HPV vaccine doses in our sample during the reference period.	Secondary data from the immunization program
Session-based adjustment	Proportion of vaccination sessions delivering HPV1 among all HPV vaccination sessions in our sample during the reference period. This adjustment factor was used only at the health facility level.	Primary data extracted at health facilities
Quantity-based adjustment	The estimated quantity of HPV1 delivered among the number of all routine immunization program vaccines during the reference period.	Secondary data from the immunization program
Volume-based adjustment	The volume in cubic centimeters (cm^3^) of HPV1 delivered among the volume of all routine immunization program vaccines during the reference period.	Secondary data from the immunization program
Frequency-based adjustment	Divided by two when activity was reported in the interviews as done “once per dose administration” or for both “dose 1” and “dose 2.” Eliminated occurrence when activity was reported as done for HPV2 only.	Primary data from questionnaire responses

HPV = human papillomavirus; HPV1 = HPV vaccine first dose; HPV2 = HPV vaccine second dose.

We conducted a 1-way sensitivity analysis (OWSA) on the financial and economic cost per dose and per adolescent receiving the single-dose schedule (excluding vaccine and supplies procurement costs) by individually varying each of the adjustment factors used in the analysis. We held constant the inputs and resultant costs for delivering the full two-dose schedule. For the OWSA, we varied the adjustment factors described in Table 1, as they are the underlying assumptions of how delivery costs would shift under a hypothetical single-dose schedule. Low and high values were evaluated for the dose-based adjustment factor assuming that 50% and 70% of all doses delivered under a two-dose schedule were HPV1, respectively. Similarly, low and high values for the session-based adjustment factor assumed 50% and 100% of HPV vaccine sessions conducted with the two-dose schedule were used to deliver HPV1, respectively. For the quantity- and volume-based adjustments, the low value assumed that the proportion of HPV1 vaccines stored was 25% less than at baseline, whereas the high value assumed 25% more HPV1 doses than at baseline. For the frequency-based adjustment factors for training and social mobilization activities, the low value assumed that 50% of activities conducted under the two-dose schedule were focused on HPV1, whereas the high value assumed that all activities (100%) were done for HPV1. Input values for the OWSA are shown in Supplementary Table 2 (available online).

## Results

### Changes to service delivery and mean costs at health facility level

Under a two-dose schedule, the average number of vaccination sessions conducted ranged from 4.0 (Ethiopia) to 25.6 (Sri Lanka) per year (Table 3) (13). In Rwanda and Uganda, the number of sessions conducted under a hypothetical single-dose schedule decreased by approximately half, because HPV1 and HPV2 were delivered separately in these countries. In the other countries, the number of sessions conducted decreased between 11% (Guyana) and 28% (Sri Lanka), due to a greater number of sessions delivering both doses together. The average number of doses delivered at each vaccinating health facility ranged from 170 (Guyana) to 761 (Sri Lanka) with the two-dose schedule and decreased between 31% (Guyana) and 47% (Rwanda) under a single-dose schedule.

**Table 3.** Average number of sessions and doses delivered per vaccinating health facility under a two-dose and hypothetical single-dose schedule

	Ethiopia		Guyana		Rwanda		Sri Lanka		Uganda	
	Sessions	Doses	Sessions	Doses	Sessions	Doses	Sessions	Doses	Sessions	Doses
2-dose schedule	4.0	411	5.4	170	9.5	613	25.6	761	6.4	226
1-dose schedule	3.6	253	4.1	117	4.7	323	18.5	488	3.2	144
% change	−11%	−38.5%	−23%	−31%	−51%	−47%	−28%	−36%	−50%	−36%

The estimated financial costs for HPV vaccine delivery under the two- and single-dose schedules exclude the costs of vaccines and syringes. Under a single-dose schedule, the modeled reductions to mean financial costs ranged from 11% in Ethiopia to 46% in Rwanda (Supplementary Table 3, available online). Activities representing a large share of mean financial costs included service delivery in all countries, vaccine collection or distribution and storage in Guyana and Rwanda, social mobilization in Ethiopia and Uganda, and training in Guyana and Uganda. Estimated cost savings for these activities with a single-dose schedule ranged from 11% (Ethiopia) to 51% (Rwanda) for service delivery, 22% (Guyana) to 43% (Rwanda) for vaccine collection or distribution and storage, no change (Ethiopia) to 50% (Sri Lanka) for social mobilization, and no changes to training (Supplementary Table 3, available online). Proportional reductions in mean economic delivery costs at the health facility level were in the same ranges as for financial cost (Supplementary Table 4, available online).

### Mean costs at administrative levels

Estimated financial costs under two- and single-dose schedules at subnational administrative levels are shown in Supplementary Table 3 (available online). Decreases to mean financial costs under a hypothetical single-dose schedule ranged from 11% (Uganda) to 34% (Rwanda) at the district level and 16% (Guyana) to 37% (Ethiopia) at the regional level. At the district level in all countries and at the regional level in Guyana, vaccine collection or distribution and storage represented a large share of mean financial costs. Reductions to this activity under a single-dose schedule ranged from 22% (Guyana) to 39% (Rwanda) (Supplementary Table 3, available online). Other activities accounting for a large share of financial costs at the district level were program planning and supervision in Ethiopia, Rwanda, and Uganda. Under modeled assumptions, there was no change to costs for program planning, and proportional savings for supervision ranged from minimal to none (Ethiopia and Uganda) to 39% (Rwanda) under a single-dose schedule (Supplementary Table 3, available online).

For economic costs, health worker time accounted for 20% or more of mean economic costs at the subnational administrative level in Guyana, Rwanda, and Sri Lanka (Supplementary Table 4, available online). In these countries, estimated decreases within this cost type ranged from 31% (Guyana) to 50% (Rwanda) with a single-dose schedule.

National-level financial costs for vaccine delivery under two- and single-dose schedules are described in Supplementary Table 3 (available online). In absolute terms, the estimated savings in financial costs with a hypothetical single-dose schedule were largest in Uganda at $88 321, which had the largest financial costs under a two-dose schedule. Proportional savings were highest in Rwanda at 40% and lowest in Guyana at 2%, based on our model. Social mobilization accounted for a large proportion of delivery costs at the national level in Ethiopia, Rwanda, and Uganda; among these countries, the proportional decreases estimated for this activity were 12% (Ethiopia) and 50% (Rwanda and Uganda) (Supplementary Table 3). In Rwanda and Sri Lanka, vaccine procurement—which only includes the costs of shipping, handling, and customs clearance for vaccines and supplies—was a key contributor to financial costs at the national level and decreased by 36% (Sri Lanka) and 49% (Rwanda) (Supplemental Table 3). Estimated economic costs followed a similar pattern. Under a single-dose schedule, Uganda had the highest absolute savings and Rwanda had the greatest proportional savings for economic costs (Supplemental Table 4).

### Cost per dose and for delivery of full vaccination schedule

Under a two-dose schedule, the cost per dose aggregated across all levels of the health system ranged from $0.27 (Sri Lanka) to $3.32 (Uganda) for financial costs and $3.09 (Rwanda) to $17.20 (Guyana) for economic costs (Table 4) (13). With a hypothetical single-dose schedule, financial costs per dose increased and ranged from $0.29 (Sri Lanka) to $3.94 (Uganda). The proportional increase ranged between 9% (Sri Lanka) and 41% (Ethiopia) (Supplemental Table 5). The estimated economic cost per dose for a single-dose schedule ranged from $3.29 (Rwanda) to $18.92 (Guyana), with the proportional increase in cost per dose between the schedules ranging from 4% (Uganda) to 34% (Ethiopia) (Supplemental Table 5).

**Table 4.** Cost per dose estimates for delivering a two-dose and hypothetical single-dose schedule, excluding the cost of vaccines and supplies (2019 US dollars)

		Ethiopia		Guyana		Rwanda		Sri Lanka		Uganda	
		2-dose	1-dose	2-dose	1-dose	2-dose	1-dose	2-dose	1-dose	2-dose	1-dose
Mean costs per dose	Financial	$2.23	$3.15	$2.10	$2.72	$1.03	$1.15	$0.27	$0.29	$3.32	$3.94
	Economic	$7.19	$9.62	$17.20	$18.92	$3.09	$3.29	$3.88	$4.34	$7.58	$7.90
Mean costs per adolescent receiving full schedule	Financial	$4.46	$3.15	$4.20	$2.72	$2.06	$1.15	$0.54	$0.29	$6.64	$3.94
	Economic	$14.38	$9.62	$34.40	$18.92	$6.18	$3.29	$7.76	$4.34	$15.16	$7.90

The estimated cost per adolescent receiving the full vaccination schedule is described in Table 4. For a two-dose schedule, these estimates ranged from $0.54 (Sri Lanka) to $6.64 (Uganda) for financial costs and $6.18 (Rwanda) to $34.40 (Guyana) for economic costs. For a single-dose schedule, these estimates were the same as the cost per dose. When compared with a two-dose schedule, the cost per adolescent receiving the full vaccination schedule decreased between 29% (Ethiopia) and 45% (Sri Lanka) for financial costs, and between 35% (Ethiopia) to 48% (Sri Lanka) for economic costs (Supplementary Table 5, available online).

### Vaccine and syringe procurement costs

The estimated amount spent on vaccine and syringe procurement costs for the cohort vaccinated under the two-dose schedule ranged from $283 581 (Guyana) to $10.4 million (Ethiopia) (Table 5). Financial cost savings for procurement under a single-dose schedule ranged from $88 000 (Guyana) to $4.6 million (Ethiopia). In terms of proportional reductions, these were highest in Rwanda (49%) and lowest in Guyana (31%).

**Table 5.** Estimated national level financial procurement costs for vaccines and syringes under a two-dose and hypothetical single-dose schedule (2019 US dollars)

	Ethiopia	Guyana	Rwanda	Sri Lanka	Uganda
2-dose schedule	$10 399 067	$283 581	$1 337 345	$1 432 167	$4 727 039
1-dose schedule	$5 781 840	$195 557	$679 371	$917 733	$3 006 397
% change	−44%	−31%	−49%	−36%	−36%

### Reductions in delivery and vaccine procurement costs

**Figure 1.**
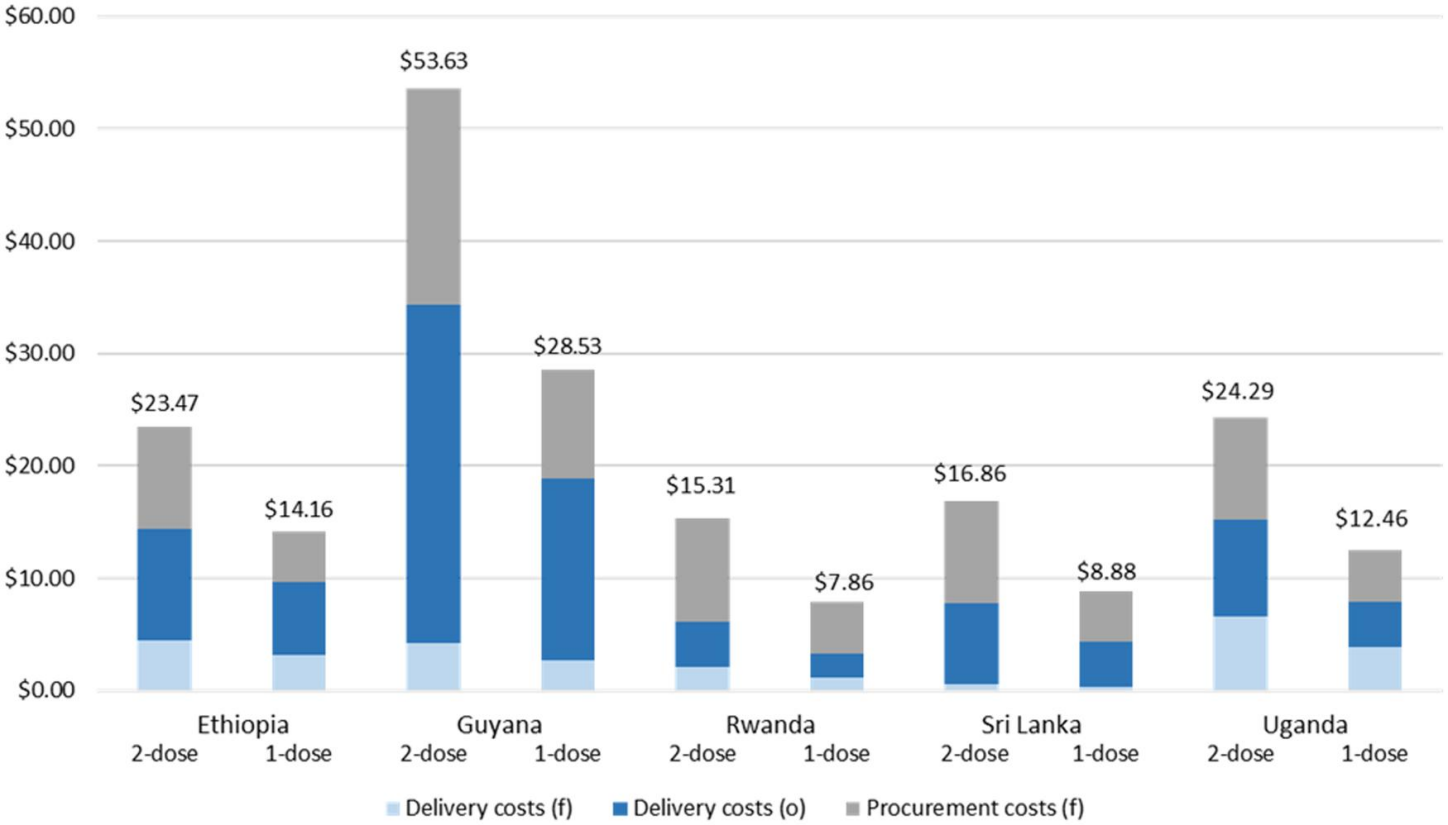
Delivery and procurement costs per adolescent to deliver the full HPV vaccination schedule under a two-dose and hypothetical single-dose schedule (2019 US dollars). Economic costs are the sum of financial and opportunity costs. f = financial costs; HPV = human papillomavirus; o = opportunity costs.

When we combined the vaccine delivery and procurement costs per adolescent receiving the full vaccination schedule, we observed decreases of at least 40% in all countries when moving from a two- to a single-dose schedule (Figure 1). For financial costs, these estimates ranged from $9.64 (Sri Lanka) to $23.43 (Guyana) for a two-dose schedule and $4.84 (Sri Lanka) to $12.34 (Guyana) for a single-dose schedule. For economic costs, this range was $15.31 (Rwanda) to $53.63 (Guyana) for the two-dose schedule and $7.86 (Rwanda) to $28.53 (Guyana) for the single-dose schedule.

### One-way sensitivity analysis

The results from our OWSA are shown in Table 6. When holding constant the costs to deliver the two-dose schedule and varying the adjustment factors used to estimate the single-dose schedule costs, we found that the financial and economic cost per adolescent receiving the single-dose schedule always remained less costly than for a two-dose schedule across all countries, at all ranges of the adjustment factors evaluated. The adjustment factors with the greatest impact on the estimated financial and economic cost per dose and per adolescent receiving the single-dose schedule were the dose- and session-based inputs (Table 6). Under our low value input for the dose-based adjustment factor, the estimated economic cost per dose and per adolescent receiving the single-dose schedule increased the most in Sri Lanka (26%) at $5.46 compared with $4.34 at baseline. At our high value assumption for the dose-based adjustment factor, the economic cost per dose and per adolescent decreased between 17% and 27% compared with baseline estimates across the 5 countries. When varying the session-based adjustment factor, at the low value, the estimated economic cost per dose and per adolescent receiving the single-dose schedule decreased by up to 23% (Sri Lanka), whereas at the high value, the estimated economic costs increased by up to 67% (Sri Lanka). The frequency-based adjustment factors are influential in countries where training and social mobilization represented a greater share of economic delivery costs, such as in Uganda. Varying the quantity- and volume-based adjustment factors had very little impact on the single-dose cost estimates.

**Table 6.** Financial and economic cost per adolescent receiving the single-dose schedule under baseline assumptions and a 1-way sensitivity analysis of low and high values for the adjustment factors (2019 US dollars)^a^

	Ethiopia			Guyana			Rwanda			Sri Lanka			Uganda		
Adjustment factors	Baseline	Low value	High value	Baseline	Low value	High value	Baseline	Low value	High value	Baseline	Low value	High value	Baseline	Low value	High value
**Financial costs**															
Dose-based	$3.15	$3.64	$2.67	$2.72	$3.67	$2.68	$1.15	$1.18	$0.93	$0.29	$0.37	$0.27	$3.94	$4.92	$3.60
Session-based		$2.68	$3.28		$2.62	$2.81		$1.15	$1.51		$0.22	$0.39		$3.94	$4.40
Quantity-based		$3.15	$3.15		$2.72	$2.72		$1.15	$1.15		$0.29	$0.29		$3.94	$3.94
Volume-based		$3.15	$3.15		$2.61	$2.83		$1.13	$1.16		$0.29	$0.30		$3.92	$3.95
Frequency-based (Training)		$3.10	$3.43		$2.02	$2.72		$1.15	$1.15		$0.29	$0.29		$3.47	$3.94
Frequency-based (Social Mobilization)		$2.98	$3.19		$2.72	$2.72		$1.15	$1.34		$0.29	$0.29		$3.90	$4.57
**Economic costs**															
Dose-based	$9.62	$11.17	$8.47	$18.92	$22.57	$18.77	$3.29	$3.41	$2.68	$4.34	$5.46	$4.00	$7.90	$9.63	$7.31
Session-based		$8.61	$9.89		$17.78	$19.90		$3.31	$4.78		$3.35	$5.58		$7.89	$9.20
Quantity-based		$9.62	$9.62		$18.92	$18.92		$3.29	$3.29		$4.34	$4.34		$7.90	$7.90
Volume-based		$9.54	$9.69		$18.28	$19.55		$3.22	$3.36		$4.31	$4.36		$7.81	$7.98
Frequency-based (Training)		$9.09	$10.49		$17.99	$18.92		$3.29	$3.29		$4.56	$4.34		$7.09	$7.90
Frequency-based (Social Mobilization)		$9.06	$9.68		$18.85	$19.04		$3.41	$2.68		$4.16	$4.56		$7.78	$9.04

a The cost per adolescent receiving the full single-dose schedule is equivalent to the cost per dose under a single-dose schedule.

## Discussion

This modeling study explored the potential savings to delivery costs and vaccine procurement costs with a single-dose HPV vaccination schedule in 5 countries beyond the introduction years, using costing and operational research data collected for the reference year of 2019 when a two-dose schedule was implemented. In all countries evaluated, the financial and economic cost per dose increased under a single-dose schedule, whereas the vaccine delivery and procurement costs per adolescent receiving the full schedule decreased between 40% and 49% for economic costs. The increase in cost per dose can be attributed to the numerator (activities and, therefore, costs) decreasing less significantly than the denominator (number of doses delivered). For example, financial costs for training would decrease only when these activities were reported as done separately for HPV2.

Our findings show that in a country such as Rwanda, where the number of HPV1 doses delivered was nearly equivalent to the number of HPV2 doses delivered (implying similar coverage for HPV1 and HPV2), the estimated vaccine and supplies procurement costs for a single-dose schedule were approximately 50% of the procurement costs under a two-dose schedule. However, in a country such as Guyana, where substantially fewer HPV2 doses were delivered compared with HPV1 (implying a lower HPV2 coverage), the procurement costs decreased by less than 50% between the two- and single-dose schedules. Although the proportion of HPV1 vs HPV2 doses delivered with a two-dose schedule directly affects the estimated procurement costs reductions with a single-dose schedule, the magnitude of the impact of this proportion on costs lessens when vaccine delivery costs are factored in, because other program contextual factors affect the delivery cost estimates.

Similarly, when we consider the number of sessions conducted at health facilities in our sample, we see greater changes with a single-dose schedule in countries where HPV1 and HPV2 were delivered separately (ie, exclusively and at two different time points in the year). For example, in Rwanda and Uganda, total sessions are anticipated to decrease by half. In countries such as Ethiopia, Guyana, and Sri Lanka, where HPV1 and HPV2 were often delivered to adolescents at joint sessions, we saw a smaller impact on the number of sessions conducted under a single-dose schedule and the associated cost reductions. Service delivery costs contribute a large share to total HPV vaccine delivery costs (8,11); therefore, the magnitude of cost savings for service delivery with a single-dose schedule will depend on how HPV1 and HPV2 are delivered and the corresponding reduction in vaccine sessions conducted.

Our OWSA showed that none of the variation in any one of the adjustment factors alone would change the overall conclusion of our analysis that across all study countries, the cost per adolescent for the single-dose schedule is always lower than the cost per adolescent with the two-dose schedule. Two adjustment factors had the greatest impact on our single-dose schedule cost estimates: the dose-based and the session-based adjustment factors. The dose-based factor had an inverse relationship to the cost per dose and per adolescent estimates, whereas the session-based factor had a direct relationship.

Regarding the session-based adjustment factor, when we assumed that half of the HPV vaccination sessions conducted under a two-dose schedule would be conducted with a single-dose schedule, this reduced the cost per dose and per adolescent receiving the single-dose schedule, because the number of sessions conducted and associated costs of conducting sessions would decrease, compared with baseline values. However, when we assumed that the same number of vaccination sessions conducted under a two-dose schedule would be conducted under a single-dose schedule, this increased the cost per dose and per adolescent receiving the single-dose schedule, compared with the baseline estimates, because costs for conducting sessions would remain the same with the single-dose schedule, whereas the denominator (number of HPV1 doses delivered) decreased. Therefore, for countries where the same number of vaccination sessions would be conducted under a single-dose schedule as under a two-dose schedule, the potential cost savings of implementing a single-dose schedule will be less substantial.

The OWSA also showed that the impact of the frequency-based adjustment factors depended on the intensity of the training and social mobilization activities carried out under a two-dose schedule. For example, we found that varying these adjustment factors had little or no impact on the estimates when the activity was not done often or at all and therefore contributed a small share to estimated baseline costs.

Considering procurement costs for vaccine product and supplies, which contribute a substantial proportion of ongoing costs for HPV vaccination programs in LMICs (10), these costs decreased more in Rwanda and Ethiopia, where HPV2 made up nearly half of doses delivered, but less in Guyana, where HPV2 represented 31% of the doses administered in 2019. As countries graduate from receiving support from Gavi, the Vaccine Alliance, and begin fully self-financing, they will bear a greater share of vaccine procurement costs and could realize greater savings over time from implementing a single-dose schedule.

The anticipated cost savings are but one programmatic benefit of a single-dose schedule. Delivering one dose per child would reduce logistical requirements to follow up children missing their second dose, reduce visits for the child (and their caregiver for facility- and community-based delivery), and offer time savings for an increasingly constrained health-care workforce.

Our findings are similar to other costing studies that assessed how dosing schedule affects HPV vaccine delivery costs. A cost of delivery study in Tanzania used modeling to estimate the delivery costs for a hypothetical single-dose schedule using data collected from sites implementing a two-dose schedule and found that costs per dose increased from $2.22 (financial cost) and $10.01 (economic cost) under a two-dose schedule to $2.51 (financial cost) and $12.18 (economic cost) under a single-dose schedule (11). However, in this same study, the cost per fully immunized child decreased 51% for financial cost and 48% for economic cost when moving from a two-dose to a single-dose schedule. The Tanzania study found that the greatest reductions to economic costs were for vaccine and injection supplies, service delivery, and cold chain-related costs, aligning with our findings. A prior costing study in Mozambique also estimated the changes in delivery costs when implementing a reduction in dose schedule, with projected annual national program costs decreasing by 50% (12). This proportion is similar to our estimate for HPV vaccine delivery and procurement costs per adolescent receiving the full schedule, which decreased between 40% and 49% for economic costs when moving from a two- to a single-dose schedule.

Our study findings can be used by stakeholders such as immunization program managers and donors to inform budgeting and planning and when making decisions regarding the cost implications of switching to a single-dose schedule. Our findings can also be used by other researchers as inputs into cost-effectiveness analyses.

Our study has several limitations. First, this is a scenario analysis using retrospective data from 2019 to estimate costs if only HPV1 was delivered during the reference period. As countries move to adopt a single-dose schedule, overall program structure may shift, and these shifts will be context-specific, but they are not accounted for in this analysis. We also did not consider the initial investment required for a schedule switch such as retraining, social mobilization, and printing of new record-keeping materials. The cost savings presented here are an estimate of what would be realized after the transition period, compared with the routine delivery of a two-dose schedule. Second, we used dose-based reductions for health worker time for all activities other than service delivery. This proportion was selected as a proxy for anticipating how time costs would be reduced in delivering a lower volume of vaccine. Closer analysis of how health workers self-reported their time could provide more precise estimates of cost savings when implementing a single-dose schedule. We also did not explore the relationship between implementing a single-dose schedule, the possible changes to coverage, and the corresponding impacts on costs.

We relied on facility records and national-level data on HPV1 and HPV2 doses administered, and if these data are incorrect, the number of HPV2 doses could be an overestimate or an underestimate, affecting our results. Lastly, this work is based on our estimates of how program activities and their costs would change based on modeling. Empirical evidence from programs implementing a single-dose schedule is needed to validate these and other estimates of ongoing delivery costs.

Our findings are based on data from 5 countries and so may not be generalizable across all countries, but they are still insightful because they provide evidence that remains consistent across the 5 countries. In addition, although the data used for the analyses are from costing studies that have relatively larger sample sizes (10), larger sample sizes could provide even more robust estimates.

This study presented the estimated cost savings of implementing a single-dose HPV vaccine schedule in 5 countries compared with a two-dose schedule. For delivery costs alone, we found that the estimated financial cost per dose could increase under a single-dose schedule, but the financial cost to deliver the full schedule per adolescent could decrease by as much as 45%. When we consider vaccine procurement and delivery costs together, the economic cost per adolescent to deliver the full schedule could decrease between 40% and 49%. A single-dose HPV vaccination schedule is likely to reduce ongoing program costs and to increase the likelihood of program affordability and sustainability.

## Supplementary Material

lgae037_Supplementary_Data

## Data Availability

The raw data used for this analysis are available on DataVerse: PATH, 2024, “HPV vaccine cost of delivery and operational context study raw data,” https://doi.org/10.7910/DVN/EVRKV3, Harvard Dataverse, v1.
