# Supplementary material for: Evaluating potential program cost savings with a single-dose HPV vaccination schedule: a modeling study
Source: J Natl Cancer Inst Monogr. 2024 Nov 12;2024(67):371–8. doi: 10.1093/jncimonographs/lgae037 (PMC11555269; doi:10.1093/jncimonographs/lgae037)
Supplement: lgae037_Supplementary_Data [file lgae037_supplementary_data.docx]

## Supplemental Appendix

Supplemental Table 1. Sample sizes by study country and health system level (11)

|  | **Ethiopia** | **Guyana** | **Rwanda** | **Sri Lanka** | **Uganda** |
| --- | --- | --- | --- | --- | --- |
| Health facilities | 60 | 43 | 42 | 30 | 66 |
| District administrative offices | 17 | n/a | 11 | 10 | 21 |
| Zonal/subcity administrative offices | 9 | n/a | n/a | n/a | n/a |
| Regional administrative offices | 3 | 5^a^ | n/a | n/a | n/a |
| National administrative office | 1 | 1 | 1 | 1 | 1 |

^a^ In Guyana, two administrative offices were interviewed in one region, resulting in five observations at the subnational level in four regions.

Supplemental Table 2. Input costs and adjustment factors used for baseline analysis and ranges used in the one-way sensitivity analysis

| **Input** | **Ethiopia** | **Guyana** | **Rwanda** | **Sri Lanka** | **Uganda** | **OWSA** |
| --- | --- | --- | --- | --- | --- | --- |
| *Unit prices (2019 US$)* |  |  |  |  |  |  |
| Vaccine unit price | $4.50 (Gavi) | $9.58 (PAHO Revolving Fund 2019 price for Gardasil-4) | $4.50 (Gavi) | $4.50 (Sri Lanka MOH) | $4.50 (Gavi) | n/a |
| Syringe unit price^a^ | $0.06 (UNICEF) | $0.032 (PAHO Revolving Fund) | $0.06 (UNICEF) | $0.04 (Sri Lanka MOH) | $0.06 (UNICEF) | n/a |
| *Adjustment factors* | *Baseline values* | *Baseline values* | *Baseline values* | *Baseline values* | *Baseline values* | *Low/high values, all countries* |
| Dose-based (mean and range)^b^ |  |  |  |  |  |  |
| Health facility level | 0.6153 | 0.6896 | 0.5273 | 0.6408 | 0.636 | 0.50/0.70 |
| Subnational administrative level | 0.590 (0.10–0.95) | 0.6896 | 0.506 (0.5–0.528) | 0.6408 | 0.636 | 0.50/0.70 |
| National administrative level | 0.556 | 0.6896 | 0.508 | 0.6408 | 0.636 | 0.50/0.70 |
| Session-based, health facility level only^c^ | 0.89242 | 0.7685 | 0.4949 | 0.7217 | 0.5013 | 0.50/1.0 |
| Quantity-based (mean and range)^d^ |  |  |  |  |  |  |
| Health facility level | 0.037 (0.006–0.092) | 0.1563 | 0.0238 (0.0132–0.0302) | 0.0443 | 0.0118 (0.005–0.018) | -25%/+25% |
| Subnational administrative level | 0.021 (0.003–0.065) | 0.1563 | 0.0238 (0.0132–0.0302) | 0.0303 (0.0242–0.0358) | 0.0118 (0.005–0.018) | -25%/+25% |
| National administrative level | 0.018 | 0.1563 | 0.0254 | 0.0317 | 0.012 | -25%/+25% |
| Volume-based (mean and range)^e^ |  |  |  |  |  |  |
| Health facility level | 0.116 (0.026–0.266) | 0.3888 | 0.0823 (0.0473–0.1044) | 0.2038 | 0.062 (0.026–0.093) | -25%/+25% |
| Subnational administrative level | 0.068 (0.014–0.198) | 0.3888 | 0.0823 (0.0473–0.1044) | 0.1498 (0.1269–0.1733) | 0.062 (0.026–0.093) | -25%/+25% |
| National administrative level | 0.063 | 0.3888 | 0.0831 | 0.1547 | 0.062 | -25%/+25% |
| Frequency-based^e^ |  |  |  |  |  |  |
| Health facility level | SM range: 0.5–1  TR range: 0.5–1 | SM range: 0.5–1  TR range: 0.5–1 | SM range: 0.5–1  TR range: 0.5–1 | SM range: 0–1  TR range: 1 | SM range: 0.5–1  TR range: 0.5–1 | 0.5/1.0 |
| Subnational administrative levels | SM range: 0.5–1  TR range: 0.5–1 | SM range: 0.5–1  TR range: 1 | SM range: 0.5  TR range: n/a | SM range: 1  TR range: n/a | SM range: 0.5–1  TR range: 0.5–1 | 0.5/1.0 |
| National administrative level | SM range: 0.5  TR range: 1 | SM range: 0.5  TR range: 0.5–1 | SM range: 0.5  TR range: n/a | SM range: n/a  TR range: n/a | SM range: 0.5–1  TR range: 0.5–1 | 0.5/1.0 |

^a^ Assumes 10% wastage rate when calculating total number of syringes.

^b^ For dose-based adjustments, the low value used in the OWSA assumed 50% of the HPV vaccine doses delivered under a two-dose schedule would be HPV1 doses, thus reducing the number of doses administered under a single-dose schedule compared to baseline. We anticipate this would increase the cost per dose with a single-dose schedule compared the baseline single-dose cost estimates as the denominator (number of doses delivered) is smaller. The high value for the OWSA assumed that 70% of the doses administered in a two-dose schedule would be HPV1 doses and would result in a lower cost per dose than at baseline single-dose cost estimates.

^c^ For session-based adjustments, the low value used in the OWSA assumed 50% of the HPV vaccination sessions conducted under a two-dose schedule would be conducted under a single-dose schedule. Compared to the baseline single-dose cost estimates, we anticipate this would decrease the cost per dose in countries where >50% of vaccination sessions delivered HPV1. The high value for the OWSA assumed that 100% of the sessions conducted under a two-dose schedule would still be conducted under a single-dose schedule. We anticipate this would increase the cost per dose for the single-dose schedule, compared to baseline for the single-dose schedule.

^d^ For the quantity- and volume-based adjustment factors, the low value for the OWSA assumed that the proportion of HPV1 vaccines stored was 25% less than at baseline, resulting in a smaller cost allocation to HPV vaccines and likely reducing costs, compared to baseline. For the high value used in the OWSA, we assumed the proportion of HPV1 doses stored to be 25% more, likely increasing costs compared to baseline.

^e^ For some activities and countries, only a point estimate is relevant for baseline values based on the number of activities reported. Activities were not carried out in all countries or at all levels of the health system, therefore some baseline data points are not applicable (n/a). For the frequency-based adjustment factors for training and social mobilization activities, the low value for the OWSA assumed that 50% of activities conducted under the two-dose schedule were focused on HPV1. We anticipate this would decrease the cost per dose compared to baseline estimate for the single dose schedule in countries where >50% of the activities were done for only HPV1. For the high value, we assumed all activities were done for HPV1, likely increasing the cost per dose compared to baseline values.

Abbreviations: OWSA, one-way sensitivity analysis; SM, social mobilization; TR, training; n/a, not applicable.

Supplemental Table 3. Estimated mean financial costs for HPV vaccine delivery by health system level under a two-dose and hypothetical single-dose schedule (2019 US$)

|  | Ethiopia | | | Guyana | | | Rwanda | | | Sri Lanka | | | Uganda | | |
| --- | --- | --- | --- | --- | --- | --- | --- | --- | --- | --- | --- | --- | --- | --- | --- |
| **Health facility level** | 2-dose | 1-dose | % change | 2-dose | 1-dose | % change | 2-dose | 1-dose | % change | 2-dose | 1-dose | % change | 2-dose | 1-dose | % change |
| Vaccine procurement | $0.83 | $0.51 | -38% | $0.00 | $0.00 | n/a | $10.83 | $5.71 | -47% | $0.00 | $0.00 | n/a | $3.94 | $2.51 | -36% |
| Estimating demand | $0.00 | $0.00 | n/a | $0.00 | $0.00 | n/a | $0.00 | $0.00 | n/a | $0.00 | $0.00 | n/a | $0.00 | $0.00 | n/a |
| Program planning | $10.20 | $10.20 | 0% | $17.96 | $17.96 | 0% | $9.24 | $9.24 | 0% | $0.00 | $0.00 | 0% | $61.36 | $61.36 | 0% |
| Social mobilization | $75.32 | $75.32 | 0% | $1.26 | $0.81 | -35% | $1.57 | $0.83 | -47% | $0.26 | $0.13 | -50% | $126.69 | $68.36 | -46% |
| Training | $16.47 | $16.47 | 0% | $28.64 | $28.64 | 0% | $0.32 | $0.32 | 0% | $0.00 | $0.00 | n/a | $95.14 | $94.76 | 0% |
| Crisis management | $14.73 | $9.06 | -38% | $0.65 | $0.45 | -31% | $0.00 | $0.00 | n/a | $0.00 | $0.00 | n/a | $0.96 | $0.61 | -36% |
| Vaccine delivery and storage | $35.49 | $23.69 | -33% | $75.01 | $58.21 | -22% | $69.65 | $39.64 | -43% | $5.37 | $3.46 | -36% | $29.01 | $19.82 | -32% |
| Waste management | $0.40 | $0.25 | -38% | $1.16 | $0.80 | -31% | $0.66 | $0.35 | -47% | $0.03 | $0.02 | -36% | $3.21 | $2.04 | -36% |
| Service delivery | $265.75 | $237.16 | -11% | $34.05 | $26.17 | -23% | $125.82 | $62.27 | -51% | $183.43 | $132.38 | -28% | $91.37 | $51.38 | -44% |
| Record keeping | $1.48 | $1.32 | -11% | $5.67 | $4.36 | -23% | $0.35 | $0.17 | -51% | $0.00 | $0.00 | n/a | $0.00 | $0.00 | 0% |
| Health facility level total | $420.67 | $373.98 | -11% | $164.40 | $137.39 | -16% | $218.44 | $118.53 | -46% | $189.09 | $135.99 | -28% | $411.68 | $300.84 | -27% |
| **District level** |  |  |  |  |  |  |  |  |  |  |  |  |  |  |  |
| Vaccine procurement | $0.00 | $0.00 | n/a | n/a | n/a | n/a | $0.00 | $0.00 | n/a | $0.00 | $0.00 | n/a | $0.00 | $0.00 | n/a |
| Estimating demand | $0.00 | $0.00 | n/a | n/a | n/a | n/a | $0.00 | $0.00 | n/a | $0.00 | $0.00 | n/a | $0.00 | $0.00 | n/a |
| Program planning | $140.01 | $140.01 | 0% | n/a | n/a | n/a | $40.44 | $40.44 | 0% | $16.30 | $16.30 | 0% | $1,237 | $1,237 | 0% |
| Social mobilization | $31.53 | $31.53 | 0% | n/a | n/a | n/a | $0.00 | $0.00 | n/a | $0.00 | $0.00 | n/a | $1,898 | $1,525 | -20% |
| Training | $9.47 | $6.53 | -31% | n/a | n/a | n/a | $0.00 | $0.00 | n/a | $0.00 | $0.00 | n/a | $450.01 | $450.01 | 0% |
| Crisis management | $9.19 | $4.83 | -47% | n/a | n/a | n/a | $0.00 | $0.00 | n/a | $0.00 | $0.00 | n/a | $12.63 | $8.03 | -36% |
| Vaccine delivery and storage | $238.98 | $162.84 | -32% | n/a | n/a | n/a | $185.01 | $112.13 | -39% | $442.33 | $295.91 | -33% | $1,847 | $1,175 | -36% |
| Waste management | $65.29 | $54.92 | -16% | n/a | n/a | n/a | $0.57 | $0.00 | -100% | $0.00 | $0.00 | n/a | $15.49 | $9.85 | -36% |
| Supervision | $159.41 | $156.23 | -2% | n/a | n/a | n/a | $78.28 | $47.45 | -39% | $0.00 | $0.00 | n/a | $3,828 | $3,825 | 0% |
| Record keeping | $23.63 | $13.93 | -41% | n/a | n/a | n/a | $0.00 | $0.00 | n/a | $0.59 | $0.43 | -28% | $7.71 | $2.46 | -68% |
| District level total | $677.51 | $570.82 | -16% | n/a | n/a | n/a | $304.31 | $200.03 | -34% | $459.22 | $312.63 | -32% | $9,296 | $8,233 | -11% |
| **Zonal/subcity level** |  |  |  |  |  |  |  |  |  |  |  |  |  |  |  |
| Vaccine procurement | $0.00 | $0.00 | n/a | n/a | n/a | n/a | n/a | n/a | n/a | n/a | n/a | n/a | n/a | n/a | n/a |
| Estimating demand | $0.00 | $0.00 | n/a | n/a | n/a | n/a | n/a | n/a | n/a | n/a | n/a | n/a | n/a | n/a | n/a |
| Program planning | $7,291 | $7,291 | 0% | n/a | n/a | n/a | n/a | n/a | n/a | n/a | n/a | n/a | n/a | n/a | n/a |
| Social mobilization | $0.00 | $0.00 | n/a | n/a | n/a | n/a | n/a | n/a | n/a | n/a | n/a | n/a | n/a | n/a | n/a |
| Training | $5,736 | $3,043 | -47% | n/a | n/a | n/a | n/a | n/a | n/a | n/a | n/a | n/a | n/a | n/a | n/a |
| Crisis management | $9.38 | $5.11 | -46% | n/a | n/a | n/a | n/a | n/a | n/a | n/a | n/a | n/a | n/a | n/a | n/a |
| Vaccine delivery and storage | $571.41 | $340.19 | -40% | n/a | n/a | n/a | n/a | n/a | n/a | n/a | n/a | n/a | n/a | n/a | n/a |
| Waste management | $0.00 | $0.00 | n/a | n/a | n/a | n/a | n/a | n/a | n/a | n/a | n/a | n/a | n/a | n/a | n/a |
| Supervision | $1,866 | $2,065 | 11% | n/a | n/a | n/a | n/a | n/a | n/a | n/a | n/a | n/a | n/a | n/a | n/a |
| Record keeping | $29.52 | $17.77 | -40% | n/a | n/a | n/a | n/a | n/a | n/a | n/a | n/a | n/a | n/a | n/a | n/a |
| Zonal/subcity level total | $15,503 | $12,762 | -18% | n/a | n/a | n/a | n/a | n/a | n/a | n/a | n/a | n/a | n/a | n/a | n/a |
| **Regional level** |  |  |  |  |  |  |  |  |  |  |  |  |  |  |  |
| Vaccine procurement | $0.00 | $0.00 | n/a | $0.00 | $0.00 | n/a | n/a | n/a | n/a | n/a | n/a | n/a | n/a | n/a | n/a |
| Estimating demand | $0.00 | $0.00 | n/a | $0.00 | $0.00 | n/a | n/a | n/a | n/a | n/a | n/a | n/a | n/a | n/a | n/a |
| Program planning | $7,651 | $7,651 | 0% | $163.07 | $163.07 | 0% | n/a | n/a | n/a | n/a | n/a | n/a | n/a | n/a | n/a |
| Social mobilization | $9,498 | $4,749 | -50% | $163.07 | $163.07 | 0% | n/a | n/a | n/a | n/a | n/a | n/a | n/a | n/a | n/a |
| Training | $22,422 | $11,211 | -50% | $0.00 | $0.00 | n/a | n/a | n/a | n/a | n/a | n/a | n/a | n/a | n/a | n/a |
| Crisis management | $0.00 | $0.00 | n/a | $0.00 | $0.00 | n/a | n/a | n/a | n/a | n/a | n/a | n/a | n/a | n/a | n/a |
| Vaccine delivery and storage | $1,235 | $775.76 | -37% | $608.74 | $476.41 | -22% | n/a | n/a | n/a | n/a | n/a | n/a | n/a | n/a | n/a |
| Waste management | $0.00 | $0.00 | n/a | $81.49 | $56.19 | -31% | n/a | n/a | n/a | n/a | n/a | n/a | n/a | n/a | n/a |
| Supervision | $3,786 | $3,705 | -2% | $0.00 | $0.00 | n/a | n/a | n/a | n/a | n/a | n/a | n/a | n/a | n/a | n/a |
| Record keeping | $0.00 | $0.00 | n/a | $0.00 | $0.00 | n/a | n/a | n/a | n/a | n/a | n/a | n/a | n/a | n/a | n/a |
| Regional level total | $44,592 | $28,092 | -37% | $1,016 | $858.74 | -16% | n/a | n/a | n/a | n/a | n/a | n/a | n/a | n/a | n/a |
| **National level** |  |  |  |  |  |  |  |  |  |  |  |  |  |  |  |
| Vaccine procurement | $0.00 | $0.00 | n/a | $576.45 | $397.52 | -31% | $66,657 | $33,862 | -49% | $443.10 | $283.94 | -36% | $46,101 | $29,320 | -36% |
| Estimating demand | $0.00 | $0.00 | n/a | $0.00 | $0.00 | n/a | $0.00 | $0.00 | n/a | $0.00 | $0.00 | n/a | $0.00 | $0.00 | n/a |
| Program planning | $16,188 | $16,188 | 0% | $47.00 | $47.00 | 0% | $20,098 | $20,098 | 0% | $0.00 | $0.00 | n/a | $14,712 | $14,712 | 0% |
| Social mobilization | $33,942 | $29,920 | -12% | $8.63 | $4.32 | -50% | $55,596 | $27,798 | -50% | $0.00 | $0.00 | n/a | $140,350 | $70,175 | -50% |
| Training | $0.00 | $0.00 | n/a | $23,330 | $23,265 | 0% | $0.00 | $0.00 | n/a | $0.00 | $0.00 | n/a | $66,332 | $66,332 | 0% |
| Crisis management | $19,592 | $10,893 | -44% | $0.00 | $0.00 | n/a | $0.00 | $0.00 | n/a | $0.00 | $0.00 | n/a | $0.00 | $0.00 | n/a |
| Vaccine delivery and storage | $2,880 | $1,602 | -44% | $1,021 | $683.81 | -33% | $820.46 | $913.95 | 11% | $1,121 | $718.33 | -36% | $3,691 | $2,326 | -37% |
| Waste management | $0.00 | $0.00 | n/a | $0.00 | $0.00 | n/a | $0.00 | $0.00 | n/a | $0.00 | $0.00 | n/a | $0.00 | $0.00 | n/a |
| Supervision | $14,149 | $11,382 | -20% | $28.78 | $28.78 | 0% | $6,694 | $6,694 | 0% | $0.00 | $0.00 | n/a | $26,350 | $26,350 | 0% |
| Record keeping | $0.00 | $0.00 | n/a | $0.00 | $0.00 | n/a | $0.00 | $0.00 | n/a | $0.00 | $0.00 | n/a | $0.00 | $0.00 | n/a |
| National level total | $86,751 | $69,985 | -19% | $25,012 | $24,426 | -2% | $149,866 | $89,366 | -40% | $1,564 | $1,002 | -36% | $297,536 | $209,215 | -30% |

Not applicable (n/a) indicates there were no costs under the two-dose schedule therefore not applicable to calculate percent change.

Supplemental Table 4. Estimated mean economic costs for HPV vaccine delivery by health system level under a two-dose and hypothetical single-dose schedule (2019 US$)

|  | **Ethiopia** | | | **Guyana** | | | **Rwanda** | | | **Sri Lanka** | | | **Uganda** | | |
| --- | --- | --- | --- | --- | --- | --- | --- | --- | --- | --- | --- | --- | --- | --- | --- |
| **Health facility level** | 2-dose | 1-dose | % change | 2-dose | 1-dose | % change | 2-dose | 1-dose | % change | 2-dose | 1-dose | % change | 2-dose | 1-dose | % change |
| Vaccine procurement | $20.26 | $12.47 | -38% | $152.74 | $105.33 | -31% | $27.63 | $14.57 | -47% | $64.26 | $41.18 | -36% | $41.72 | $26.53 | -36% |
| Estimating demand | $32.22 | $19.82 | -38% | $122.97 | $84.80 | -31% | $40.88 | $21.56 | -47% | $14.61 | $9.36 | -36% | $13.10 | $8.33 | -36% |
| Program planning | $117.18 | $108.21 | -8% | $154.10 | $129.75 | -16% | $89.38 | $68.98 | -23% | $54.09 | $37.22 | -31% | $113.96 | $103.25 | -9% |
| Social mobilization | $304.03 | $300.28 | -1% | $162.51 | $113.83 | -30% | $72.33 | $37.64 | -48% | $463.34 | $333.63 | -28% | $308.62 | $178.81 | -42% |
| Training | $295.04 | $278.69 | -6% | $102.67 | $89.54 | -13% | $3.63 | $2.06 | -43% | $17.59 | $11.27 | -36% | $186.70 | $183.59 | -2% |
| Crisis management | $49.75 | $30.61 | -38% | $183.68 | $126.66 | -31% | $1.54 | $0.81 | -47% | $15.95 | $10.22 | -36% | $16.81 | $10.69 | -36% |
| Vaccine delivery and storage | $79.82 | $64.55 | -19% | $433.61 | $339.55 | -22% | $168.53 | $111.21 | -34% | $38.82 | $24.69 | -36% | $113.72 | $74.48 | -35% |
| Waste management | $32.96 | $36.43 | 11% | $53.77 | $43.41 | -19% | $15.12 | $1.66 | -89% | $0.03 | $0.02 | -36% | $87.23 | $7.68 | -91% |
| Service delivery | $551.54 | $492.20 | -11% | $359.52 | $276.29 | -23% | $640.03 | $316.75 | -51% | $2,457 | $1,773 | -28% | $272.59 | $136.65 | -50% |
| Record keeping | $67.38 | $41.87 | -38% | $253.55 | $175.30 | -31% | $22.98 | $12.11 | -47% | $64.11 | $41.08 | -36% | $57.76 | $36.73 | -36% |
| Health facility level total | $1,550 | $1,385 | -11% | $1,979 | $1,484 | -25% | $1,082 | $587.36 | -46% | $3,190 | $2,282 | -28% | $1,212 | $766.74 | -37% |
| **District level** |  |  |  |  |  |  |  |  |  |  |  |  |  |  |  |
| Vaccine procurement | $0.00 | $0.00 | n/a | n/a | n/a | n/a | $0.00 | $0.00 | n/a | $0.00 | $0.00 | n/a | $0.00 | $0.00 | n/a |
| Estimating demand | $0.00 | $0.00 | n/a | n/a | n/a | n/a | $0.00 | $0.00 | n/a | $0.00 | $0.00 | n/a | $0.00 | $0.00 | n/a |
| Program planning | $1,031 | $1,031 | 0% | n/a | n/a | n/a | $42.73 | $42.73 | 0% | $16.30 | $16.30 | 0% | $1,344 | $1,344 | 0% |
| Social mobilization | $84.69 | $63.61 | -25% | n/a | n/a | n/a | $0.00 | $0.00 | n/a | $2.58 | $2.58 | 0% | $2,624 | $1,892 | -28% |
| Training | $1,194 | $598.81 | -50% | n/a | n/a | n/a | $0.00 | $0.00 | n/a | $0.00 | $0.00 | n/a | $450.01 | $450.01 | 0% |
| Crisis management | $1,331 | $772.79 | -42% | n/a | n/a | n/a | $0.00 | $0.00 | n/a | $0.00 | $0.00 | n/a | $15.27 | $9.71 | -36% |
| Vaccine delivery and storage | $385.23 | $264.86 | -31% | n/a | n/a | n/a | $603.20 | $364.12 | -40% | $1,416 | $1,026 | -27% | $2,599 | $1,658 | -36% |
| Waste management | $94.36 | $75.04 | -20% | n/a | n/a | n/a | $37.31 | $36.03 | -3% | $0.00 | $0.00 | n/a | $57.24 | $37.38 | -35% |
| Record keeping | $23.63 | $13.93 | -41% | n/a | n/a | n/a | $0.00 | $0.00 | n/a | $0.59 | $0.43 | -28% | $7.71 | $2.46 | -68% |
| Supervision | $163.09 | $159.91 | -2% | n/a | n/a | n/a | $81.25 | $50.42 | -38% | $0.00 | $0.00 | n/a | $3,828 | $3,825 | 0% |
| Health worker time | $526.31 | $280.80 | -47% | n/a | n/a | n/a | $512.59 | $258.24 | -50% | $371.35 | $237.96 | -36% | $82.77 | $52.64 | -36% |
| District level total | $4,834 | $3,261 | -33% | n/a | n/a | n/a | $1,277 | $751.54 | -41% | $1,807 | $1,284 | -29% | $11,007 | $9,271 | -16% |
| **Zonal/subcity level** |  |  |  |  |  |  |  |  |  |  |  |  |  |  |  |
| Vaccine procurement | $0.00 | $0.00 | n/a | n/a | n/a | n/a | n/a | n/a | n/a | n/a | n/a | n/a | n/a | n/a | n/a |
| Estimating demand | $0.00 | $0.00 | n/a | n/a | n/a | n/a | n/a | n/a | n/a | n/a | n/a | n/a | n/a | n/a | n/a |
| Program planning | $7,453 | $7,453 | 0% | n/a | n/a | n/a | n/a | n/a | n/a | n/a | n/a | n/a | n/a | n/a | n/a |
| Social mobilization | $0.00 | $0.00 | n/a | n/a | n/a | n/a | n/a | n/a | n/a | n/a | n/a | n/a | n/a | n/a | n/a |
| Training | $7,656 | $4,003 | -48% | n/a | n/a | n/a | n/a | n/a | n/a | n/a | n/a | n/a | n/a | n/a | n/a |
| Crisis management | $48.01 | $26.57 | -45% | n/a | n/a | n/a | n/a | n/a | n/a | n/a | n/a | n/a | n/a | n/a | n/a |
| Vaccine delivery and storage | $734.86 | $435.24 | -41% | n/a | n/a | n/a | n/a | n/a | n/a | n/a | n/a | n/a | n/a | n/a | n/a |
| Waste management | $0.00 | $0.00 | n/a | n/a | n/a | n/a | n/a | n/a | n/a | n/a | n/a | n/a | n/a | n/a | n/a |
| Record keeping | $29.52 | $17.77 | -40% | n/a | n/a | n/a | n/a | n/a | n/a | n/a | n/a | n/a | n/a | n/a | n/a |
| Supervision | $1,883 | $2,074 | 10% | n/a | n/a | n/a | n/a | n/a | n/a | n/a | n/a | n/a | n/a | n/a | n/a |
| Health worker time | $679.73 | $402.46 | -41% | n/a | n/a | n/a | n/a | n/a | n/a | n/a | n/a | n/a | n/a | n/a | n/a |
| Zonal/subcity level total | $18,485 | $14,412 | -22% | n/a | n/a | n/a | n/a | n/a | n/a | n/a | n/a | n/a | n/a | n/a | n/a |

|  | **Ethiopia** | | | **Guyana** | | | **Rwanda** | | | **Sri Lanka** | | | **Uganda** | | |
| --- | --- | --- | --- | --- | --- | --- | --- | --- | --- | --- | --- | --- | --- | --- | --- |
| **Regional level** | 2-dose | 1-dose | % change | 2-dose | 1-dose | 2-dose | 1-dose | % change | 2-dose | 1-dose | 2-dose | 1-dose | % change | 2-dose | 1-dose |
| Vaccine procurement | $0.00 | $0.00 | n/a | $0.00 | $0.00 | n/a | n/a | n/a | n/a | n/a | n/a | n/a | n/a | n/a | n/a |
| Estimating demand | $0.00 | $0.00 | n/a | $0.00 | $0.00 | n/a | n/a | n/a | n/a | n/a | n/a | n/a | n/a | n/a | n/a |
| Program planning | $7,822 | $7,822 | 0% | $703.23 | $703.23 | 0% | n/a | n/a | n/a | n/a | n/a | n/a | n/a | n/a | n/a |
| Social mobilization | $9,724 | $4,862 | -50% | $556.86 | $556.86 | 0% | n/a | n/a | n/a | n/a | n/a | n/a | n/a | n/a | n/a |
| Training | $31,653 | $15,827 | -50% | $555.93 | $555.93 | 0% | n/a | n/a | n/a | n/a | n/a | n/a | n/a | n/a | n/a |
| Crisis management | $0.00 | $0.00 | n/a | $2,897 | $1,998 | -31% | n/a | n/a | n/a | n/a | n/a | n/a | n/a | n/a | n/a |
| Vaccine delivery and storage | $1,481 | $929.68 | -37% | $2,095 | $1,611 | -23% | n/a | n/a | n/a | n/a | n/a | n/a | n/a | n/a | n/a |
| Waste management | $16.97 | $11.07 | -35% | $855.84 | $683.42 | -20% | n/a | n/a | n/a | n/a | n/a | n/a | n/a | n/a | n/a |
| Record keeping | $0.00 | $0.00 | n/a | $0.00 | $0.00 | n/a | n/a | n/a | n/a | n/a | n/a | n/a | n/a | n/a | n/a |
| Supervision | $7,541 | $5,588 | -26% | $0.00 | $0.00 | n/a | n/a | n/a | n/a | n/a | n/a | n/a | n/a | n/a | n/a |
| Health worker time | $365.91 | $227.02 | -38% | $4,905 | $3,383 | -31% | n/a | n/a | n/a | n/a | n/a | n/a | n/a | n/a | n/a |
| Regional level total | $58,604 | $35,267 | -40% | $12,570 | $9,492 | -24% | n/a | n/a | n/a | n/a | n/a | n/a | n/a | n/a | n/a |
| **National level** |  |  |  |  |  |  |  |  |  |  |  |  |  |  |  |
| Vaccine procurement | $0.00 | $0.00 | n/a | $576.45 | $397.52 | -31% | $66,657 | $33,862 | -49% | $443.10 | $283.94 | -36% | $46,101 | $29,320 | -36% |
| Estimating demand | $0.00 | $0.00 | n/a | $0.00 | $0.00 | n/a | $0.00 | $0.00 | n/a | $0.00 | $0.00 | n/a | $0.00 | $0.00 | n/a |
| Program planning | $16,188 | $16,188 | 0% | $1,065 | $1,065 | 0% | $20,945 | $20,945 | 0% | $0.00 | $0.00 | n/a | $15,238 | $15,238 | 0% |
| Social mobilization | $34,098 | $30,024 | -12% | $359.05 | $179.53 | -50% | $55,596 | $27,798 | -50% | $0.00 | $0.00 | n/a | $140,405 | $70,203 | -50% |
| Training | $0.00 | $0.00 | n/a | $23,330 | $23,265 | 0% | $0.00 | $0.00 | n/a | $0.00 | $0.00 | n/a | $66,332 | $66,332 | 0% |
| Crisis management | $21,202 | $11,788 | -44% | $0.00 | $0.00 | n/a | $0.00 | $0.00 | n/a | $0.00 | $0.00 | n/a | $0.00 | $0.00 | n/a |
| Vaccine delivery and storage | $5,897 | $3,367 | -43% | $2,383 | $1,787 | -25% | $4,030 | $3,980 | -1% | $5,499 | $3,593 | -35% | $9,487 | $6,061 | -36% |
| Waste management | $0.00 | $0.00 | n/a | $0.00 | $0.00 | n/a | $0.00 | $0.00 | n/a | $0.00 | $0.00 | n/a | $0.00 | $0.00 | n/a |
| Record keeping | $15,413 | $12,044 | -22% | $28.78 | $28.78 | 0% | $6,759 | $6,759 | 0% | $0.00 | $0.00 | n/a | $26,350 | $26,350 | 0% |
| Supervision | $0.00 | $0.00 | n/a | $0.00 | $0.00 | n/a | $0.00 | $0.00 | n/a | $0.00 | $0.00 | n/a | $0.00 | $0.00 | n/a |
| Health worker time | $4,042 | $2,247 | -44% | $33,201 | $22,896 | -31% | $7,231 | $3,674 | -49% | $1,714 | $1,099 | -36% | $366.18 | $232.89 | -36% |
| National level total | $96,840 | $75,659 | -22% | $60,944 | $49,619 | -19% | $161,219 | $97,018 | -40% | $7,657 | $4,976 | -35% | $304,278 | $213,736 | -30% |

Supplemental Table 5. Estimated HPV vaccine delivery cost per dose and cost per adolescent receiving the full schedule, by health system level and schedule (2019 US$)

|  | **Ethiopia** | | | **Guyana** | | | **Rwanda** | | | **Sri Lanka** | | | **Uganda** | | |
| --- | --- | --- | --- | --- | --- | --- | --- | --- | --- | --- | --- | --- | --- | --- | --- |
| **Financial cost per dose** | 2-dose schedule | 1-dose schedule | % change | 2-dose schedule | 1-dose schedule | % change | 2-dose schedule | 1-dose schedule | % change | 2-dose schedule | 1-dose schedule | % change | 2-dose schedule | 1-dose schedule | % change |
| Health facility | $1.05 | $1.52 | 45% | $1.05 | $1.27 | 21% | $0.48 | $0.49 | 3% | $0.22 | $0.25 | 12% | $2.20 | $2.46 | 12% |
| District | $0.32 | $0.46 | 44% | n/a | n/a | n/a | $0.04 | $0.05 | 33% | $0.04 | $0.04 | 7% | $0.83 | $1.16 | 40% |
| Zone/subcity | $0.59 | $0.86 | 45% | n/a | n/a | n/a | n/a | n/a | n/a | n/a | n/a | n/a | n/a | n/a | n/a |
| Regional | $0.23 | $0.25 | 10% | $0.20 | $0.25 | 23% | n/a | n/a | n/a | n/a | n/a | n/a | n/a | n/a | n/a |
| National | $0.04 | $0.06 | 38% | $0.85 | $1.20 | 41% | $0.51 | $0.60 | 18% | $0.01 | $0.00 | -50% | $0.29 | $0.32 | 10% |
| Total financial cost per dose | **$2.23** | **$3.15** | **41%** | **$2.10** | **$2.72** | **30%** | **$1.03** | **$1.15** | **11%** | **$0.27** | **$0.29** | **9%** | **$3.32** | **$3.94** | **19%** |
| **Economic cost per dose** |  |  |  |  |  |  |  |  |  |  |  |  |  |  |  |
| Health facility | $3.88 | $5.64 | 45% | $12.64 | $13.75 | 9% | $2.37 | $2.44 | 3% | $3.70 | $4.13 | 12% | $6.30 | $6.27 | -1% |
| District | $2.27 | $2.63 | 16% | n/a | n/a | n/a | $0.17 | $0.20 | 17% | $0.16 | $0.18 | 10% | $0.99 | $1.31 | 32% |
| Zone/subcity | $0.70 | $0.97 | 38% | n/a | n/a | n/a | n/a | n/a | n/a | n/a | n/a | n/a | n/a | n/a | n/a |
| Regional | $0.30 | $0.32 | 6% | $2.49 | $2.72 | 9% | n/a | n/a | n/a | n/a | n/a | n/a | n/a | n/a | n/a |
| National | $0.04 | $0.06 | 49% | $2.07 | $2.44 | 18% | $0.55 | $0.65 | 19% | $0.02 | $0.02 | 23% | $0.29 | $0.32 | 12% |
| Total economic cost per dose | **$7.19** | **$9.62** | **34%** | **$17.20** | **$18.92** | **10%** | **$3.09** | **$3.29** | **6%** | **$3.88** | **$4.34** | **12%** | **$7.58** | **$7.90** | **4%** |
| **Cost per adolescent receiving the full schedule** |  |  |  |  |  |  |  |  |  |  |  |  |  |  |  |
| Financial | $4.46 | $3.15 | -29% | $4.20 | $2.72 | -35% | $2.06 | $1.15 | -44% | $0.54 | $0.29 | -45% | $6.64 | $3.94 | -41% |
| Economic | $14.38 | $9.62 | -33% | $34.40 | $18.92 | -45% | $6.18 | $3.29 | -47% | $7.76 | $4.34 | -44% | $15.16 | $7.90 | -48% |
